# Using spatial analysis to demonstrate the heterogeneity of the cardiovascular drug-prescribing pattern in Taiwan

**DOI:** 10.1186/1471-2458-11-380

**Published:** 2011-05-24

**Authors:** Ching-Lan Cheng, Yi-Chi Chen, Tzu-Ming Liu, Yea-Huei Kao Yang

**Affiliations:** 1 Institute of Biopharmaceutical Science, College of Medicine, National Cheng Kung University, Tainan, Taiwan; 2 Department of Economics, National Cheng Kung University, Tainan, Taiwan; 3 Graduate Institute of Sustainable Tourism and Recreation Management, National Taichung University, Taichung, Taiwan; 4 Institute of Clinical Pharmacy, College of Medicine, National Cheng Kung University, Tainan, Taiwan

## Abstract

**Background:**

Geographic Information Systems (GIS) combined with spatial analytical methods could be helpful in examining patterns of drug use. Little attention has been paid to geographic variation of cardiovascular prescription use in Taiwan. The main objective was to use local spatial association statistics to test whether or not the cardiovascular medication-prescribing pattern is homogenous across 352 townships in Taiwan.

**Methods:**

The statistical methods used were the global measures of Moran's *I *and Local Indicators of Spatial Association (LISA). While Moran's *I *provides information on the overall spatial distribution of the data, LISA provides information on types of spatial association at the local level. LISA statistics can also be used to identify influential locations in spatial association analysis. The major classes of prescription cardiovascular drugs were taken from Taiwan's National Health Insurance Research Database (NHIRD), which has a coverage rate of over 97%. The dosage of each prescription was converted into defined daily doses to measure the consumption of each class of drugs. Data were analyzed with ArcGIS and GeoDa at the township level.

**Results:**

The LISA statistics showed an unusual use of cardiovascular medications in the southern townships with high local variation. Patterns of drug use also showed more low-low spatial clusters (cold spots) than high-high spatial clusters (hot spots), and those low-low associations were clustered in the rural areas.

**Conclusions:**

The cardiovascular drug prescribing patterns were heterogeneous across Taiwan. In particular, a clear pattern of north-south disparity exists. Such spatial clustering helps prioritize the target areas that require better education concerning drug use.

## Background

Spatial analysis can identify inequities in disease distribution or in resources for healthcare utilization, resulting in financial incentives in payment policies [1-3]. Spatial analysis has taken giant steps forward in the recent decade, and Geographic Information Systems (GIS) software makes it possible to undertake a sophisticated visual approach to data analysis in medical issues [4,5]. However, most studies using GIS have relied on their mapping capabilities rather than performance of statistical analyses [6-8]. Only when GIS are combined with spatial analytical methods can the result provide a helpful tool in the study of public health issues [9,10].

Spatial analyses can be compared to societal norms or objectives. The traditional descriptive statistics fail to show the complexity of interactions between individual geographic units. In contrast, spatial analysis can reveal the co-variation of properties with a geo-space, since characteristics at proximal locations tend to be correlated, either positively or negatively, which leads to spatial autocorrelation [11]. For example, a study shows that people are indirectly protected when more people in their neighborhood are vaccinated with cholera vaccine [12].

Another potential use of the spatial analysis is to examine patterns of drug use. Spatial analysis provides a method for delineating the complex nature of the associations between multiple levels of social structure and illicit drug use [13,14]. These applications have important implications in policy-making, as the associations between geography and drug use could be useful for planning drug prevention programs.

Cardiovascular disease (CVD) remains the leading cause of death and disability, representing a major burden for health systems worldwide [15,16], and impressive literature has documented geographic variation in the use of medication and procedures for treating cardiovascular disease in Western countries [17-21]. The cardiovascular medication-use patterns observed reflect the influence of a complex combination of demographic, social, economic, cultural, and environmental factors. Quality improvement programs based on geographic data may help to reduce the variation in quality of care [22].

A few previous studies have specifically examined the cardiovascular medication utilization in Taiwan [23,24]; however, seldom is the spatial pattern of drug use addressed. Therefore, the objective of this study was to investigate prescribing patterns of cardiovascular medications based on the variations of geo-space in Taiwan. We hypothesized there would be significant geographic differences in prescribing cardiovascular medications among townships.

## Methods

### Data source

We conducted a cross-sectional study by using the non-sampled National Health Insurance Research Database (NHIRD) in Taiwan. This database is population-based and derived from the claims data of the National Health Insurance program, a mandatory-enrollment and single-payer system implemented in 1995 [25]. Patients who had at least one outpatient prescription for CVD during the year 2004 were enrolled in this study. The dataset contained 22.13 million enrollees (about 97.5% of the total population in Taiwan) in 2004.

The data elements available for each patient in NHIRD contains minimal demographic characteristics, medical diagnoses (up to 5 for each admission, up to 3 for each ambulatory visit), procedures, expenditures, and all detailed prescriptions [26].

### Cardiovascular medications

The prescriptions were coded according to the Anatomical Therapeutic Chemical coding system of the NHI Pharmaceutical subsidy, which was used as the interface for retrieving the pharmaceutical claims data. Five drug classes (and ATC numbers) were recorded, including agents acting on the rennin-angiotensin system (C09), called Group A hereafter; beta blocking agents (C07), called Group B hereafter; calcium channel blockers (C08), called Group C hereafter; diuretics (C03), called Group D hereafter; and antihypertensives (C02), called Group O hereafter. We presented the proportion of use of these cardiovascular drug classes among all patients based on geographic location. We used the "defined daily dose" (DDD) as a measure unit for drug consumption, which was developed by the World Health Organization (WHO) Collaborating Center for Drug Statistics Methodology (WHO, 2004). The DDD of a pharmaceutical substance represents the assumed average maintenance dose per day for a drug, when used for its main indication in adults. For example, the recommended maintenance dose of captopril, an angiotensin converting enzyme inhibitor, for hypertension is 50 mg to 75 mg per day, the DDD of this drug was define as 50 mg from WHO. The consumption of drugs was expressed as DDDs/1000 inhabitants per day, which may serve as an estimation of the proportion of the population receiving the drug treatment. The information on residents in each township in Taiwan was based on annual statistics of the Ministry of Interior [27].

### Geographic distribution

**Figure 1:**
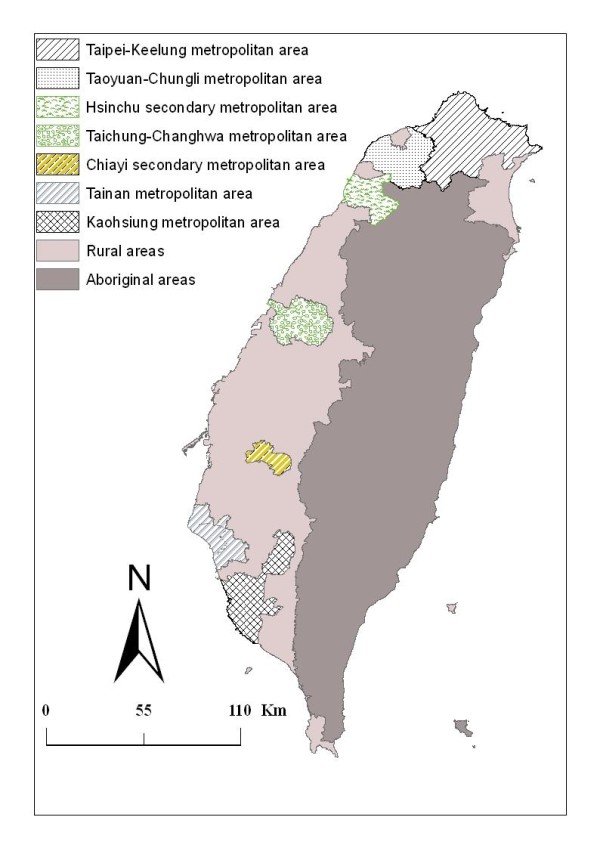
**Map of metropolitan areas and aboriginal townships in Taiwan**. The map of the study region in Taiwan includes 7 metropolitan areas and the area of 55 aboriginal townships.

Taiwan became one of East Asia's economic "Tigers" in the twentieth century. Its population was more than 22 million in the year 2000, with a combined area of approximately 36,000 square kilometers. The study area focuses on the main island of Taiwan; the offshore islands are not considered. In total, there are 352 townships included in this study, which can be further classified into 5 metropolitan areas, 2 secondary metropolitan areas, 206 rural townships, and 55 aboriginal townships (Figure 1)[28]. The metropolitan classification was revised in 1993 by the Directorate General of Budget, Accounting, and Statistics of Taiwan. According to this classification, there are 5 major metropolitan areas (Taipei-Keelung, Taoyuan-Chungli, Taichung-Changhwa, Tainan, and Kaohsiung) and 2 secondary metropolitan areas (Hsinchu and Chiayi), which consist of 66.87% of the population. Each metropolitan area usually has at least one core city with satellite cities of similar socio-economic attributes. A metropolitan area has at least 0.3 million people, with population above 1 million considered a major metropolitan area and below 1 million a secondary metropolitan area. The Council of Indigenous Peoples of Taiwan defines 55 aboriginal townships, including 30 indigenous mountain and 25 plain townships. Although Taiwan has experienced rapid economic growth, there has been a south-north (using Taichung-Changhwa as the division) inequity. That is, the region north of the Taichung-Changhwa area has a more highly developed service economy, technology complex, and financial sector than the southern region.

### Statistical Analysis

We analyzed consumption of all classes of cardiovascular medications by DDD/1000 inhabitants per day across 352 townships. We presented the descriptive statistics of drug consumption by metropolitan areas, including the mean (± standard deviation). Coefficient of variation (COV, standard deviation divided by mean) is used to describe the variations of consumption in various areas. Bivariate analysis using the *Student's t *test was conducted to compare the south and north regions of Taiwan; a two-sided *p *value < 0.05 was considered statistically significant. Data were extracted from NHIRD, and descriptive analysis were performed with SAS version 9.1 (SAS Institute Inc., Cary, North Carolina)

Local Indicators of Spatial Association (LISA) [29] allows us to decompose the study area into small locations, thus enabling the assessment of significant local spatial clustering around an individual location. In addition to the degree of spatial clustering, the detailed variations of clustering in the locally defined geo-space are identified as well as the locations of the spatial clusters. The local version of Moran's *I *at location *i *is given by:

where *n *indicates the total number of locations (352 townships), *x*_*i *_denotes the value of the variable of interest, *X*, at location *i*, *x*_*j *_denotes the observation at neighboring locations *j*, and  is the sample average of *X*. *w*_*ij *_is the so-called spatial weights matrix (connectivity matrix), which defines spatial interaction across study regions. In general, *w*_*ij *_= 1 if location *i *and location *j *are neighboring, (share a common boundary); otherwise, *w*_*ij *_= 0. In the LISA analysis, if the test statistic is not significant at any sensible level, no spatial pattern is present in the areas; i.e., observations are spatially random. When it is significant, however, two possible patterns of local spatial clusters are likely to be exhibited: (1) When *x*_*i *_is higher than the average of the entire study area () and so are its neighbors, a high-high (HH) association, a so-called hot spot, is indicated. (2) When both *x*_*i *_and its neighbors are lower than the average, the spatial tendency is low-low (LL), or a cold spot. The major limitation of Moran's *I *is that it only measures spatial clustering (spatial autocorrelation) at a global scale, but cannot detect important clusters at a local scale and determine the spatial patterns for the specific locations. To make spatial autocorrelation visually meaningful, the local Moran *I *is represented by cluster maps, in which the locations of significant spatial clusters are highlighted to identify the patterns of associations and *p *value < 0.01 was considered statistically significant.

Underlying spatial clusters can be geographic changes in risk factors, which introduce a degree of spatial autocorrelation in the outcome. In this study, spatial clusters of drug use reflect inefficiency in medical resources and utilization. Still, a variety of possible factors could lead to such inefficiency and they are location-specific. However, if there exist common factors creating diffusion or local clusters of medical inefficiency, it would be helpful and necessary to identify the locations of risk clusters and the extent of their clustering. Based on the sizes of risk areas and the significance of clusters, important risk factors shared by neighboring areas could be uncovered. Thus, from the perspective of policy making and research, priority should be given to these clustered areas.

We used the geographic information system software ArcGIS v9.2 to combine drug prescription data with Taiwan digital maps at the township level. The data was then processed by spatial analysis software GeoDa [30] to create the spatial weights matrix and to calculate the LISA value for each township.

## Results

### Geographic map and geographic distribution of drugs consumption

Figure 1 illustrates the administrative division across 352 townships in Taiwan, including 5 major metropolitan areas, 2 secondary metropolitan areas, 206 rural areas, and 55 aboriginal townships.

The pattern of drug consumption across townships indicated the highest utilization was of Group C followed by Group A, Group B, Group D, and Group O. (Table 1) Among all groups of drugs, variations in consumption by metropolitan areas were the largest in Hsinchu (COV = 100 to 119%), followed by Tainan (COV = 71 to 106%), rural township (COV = 55 to 102%) and aboriginal area (COV = 38 to 98%).

**Table 1:** The distribution of cardiovascular medications consumption by urbanicity, data presented as mean ± (SD)^a^

	Population (%)	**Group A**^**b**^	Group B	Group C	Group D	Group O
Taipei-Keelung metropolitan area (N = 44)	6,609,220 (29.4)	32.2 (42.0)	18.1 (22.1)	32.3 (43.4)	10.2 (12.8)	2.7 (4.7)
Taoyuan-Chungli metropolitan area (N = 12)	1,846,082 (8.2)	40.9 (79.5)	20.0 (34.9)	40.4 (72.0)	11.7 (23.9)	2.4 (2.8)
Hsinchu secondary metropolitan Area (N = 10)	682,132 (3.0)	18.2 (15.9)	11.0 (9.2)	21.2 (18.4)	6.1 (5.7)	1.1 (1.1)
Taichung-Changhwa metropolitan area (N = 18)	2,092,344 (9.3)	41.7 (60.3)	24.1 (33.2)	45.6 (71.5)	13.5 (20.6)	3.5 (4.4)
Chiayi secondary metropolitan area (N = 4)	373,774 (1.7)	36.5 (42.8)	19.5 (19.6)	40.1 (49.5)	13.1 (17.6)	4.3 (5.9)
Tainan metropolitan area (N = 14)	1,246,084 (5.5)	27.1 (26.7)	15.6 (14.6)	31.7 (32.9)	7.2 (7.4)	2.7 (3.8)
Kaohsiung metropolitan area (N = 28)	2,758,140 (12.2)	43.9 (94.0)	23.0 (37.8)	45.7 (82.5)	12.5 (24.6)	3.7 (4.5)
Rural townships area (N = 167)	6,100,872 (27.1)	18.2 (18.2)	10.7 (10.4)	19.3 (21.1)	5.7 (7.8)	1.6 (2.9)
Aboriginal townships area (N = 55)	812,703 (3.6)	25.3 (26.5)	10.4 (10.6)	19.6 (22.7)	7.7 (9.5)	1.3 (3.4)
Total (N = 352)	22,521,351 (100)	25.7 (40.8)	13.9 (19.1)	26.0 (40.3)	7.9 (12.7)	2.1 (3.6)

a. Data presented as DDD/1000 inhabitants per day

b. Group A, agents acting on the rennin-angiotensin system; Group B, beta blocking agents; Group C, calcium channel blockers; Group D, diuretics; Group O, antihypertensives

We did not observe any significant difference in cardiovascular medication consumption between northern and southern areas: Group A (21.2 ± 33.6, 17.8 ± 40.5; p = 0.67), Group B (11.8 ± 17.2, 10.1 ± 17.5; p = 0.51), Group C (20.8 ± 34.9, 19.0 ± 38.3; p = 0.86), Group D (6.3 ± 10.9, 5.6 ± 13.5; p = 0.83) and Group O (1.5 ± 3.1, 1.5 ± 3.4; p = 0.70), respectively.

### LISA statistics for cardiovascular drugs

**Figure 2:**
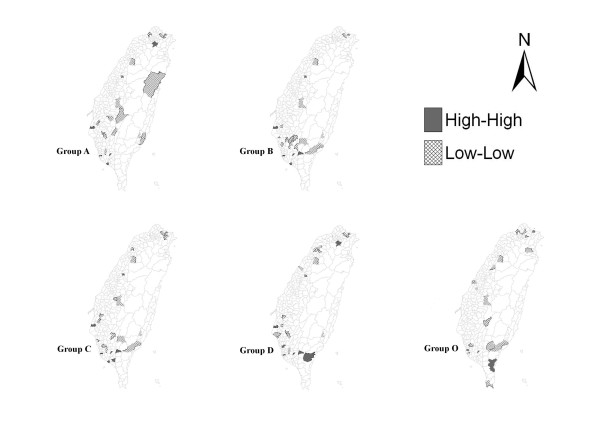
**Spatial clusters of cardiovascular drugs in Taiwan**. A high-high association of the LISA statistics indicates the location is higher than the average of the entire study area and so are its neighbors, or a hot spot; a low-low association indicates both the location and its neighbors are lower than the average, or a cold spot.

Figure 2 displays plots of the LISA statistics for each class of cardiovascular drug utilization. The marked polygons indicated areas with significant spatial clustering, while the blank polygons indicated areas with insignificant spatial clustering.

There were wide variations in utilization of all drug classes across the 352 townships in Taiwan. From the first glimpse of the LISA figures, under-utilization appeared to be the predominant spatial pattern for cardiovascular disorders across all drug classes: the total number of high-high associations (hot spots) amounts to 35, while low-low associations (cold spots) reaches to 85. Such spatial variations carried three statistical meanings: (1) variations were non-random; (2) variations were statistically significant; (3) variations exhibited effects of neighboring interactions.

### North-south discrepancy distribution

Focusing on the west of Taiwan, where more than 97 percent of the population resides, patterns of drug use exhibited more high-high associations clustered in the southern (n = 30) than northern (n = 2) townships. Thus the southern region had higher-than-average utilization of cardiovascular drugs than other parts of Taiwan. However, at the same time, the number of low-low associations also presented in southern (n = 42) more than northern areas (n = 33).

Tables 2 summarizes the distribution of spatial associations for each drug class by the levels of urbanization. Among all drug classes, a large number of significant LISA statistics were observed in metropolitan areas followed by aboriginal, rural, and secondary metropolitan areas. Among those metropolitan areas, the significant LISA statistics were mostly observed in the southern townships such as Kaohsiung and Tainan city. In other words, with each prescription drug, more observations were correlated with their neighboring values, giving evidence of possible diffusion effects of drug use.

**Table 2:** The spatial analysis of five category cardiovascular medications by urbanicity, data presented as N (%)

	**Group A**^**d**^		Group B		Group C		Group D		Group O	
					
	**H**^**a**^	**L**^**b**^	H	L	H	L	H	L	H	L
Taipei-Keelung metropolitan area (N = 44)	1 (2.3)	4 (9.1)	0 (0.0)	2 (4.5)	0 (0.0)	4 (9.1)	1 (2.3)	3 (6.8)	0 (0.0)	5 (11.4)
Hsinchu secondary metropolitan Area (N = 10)	0 (0.0)	0 (0.0)	0 (0.0)	0 (0.0)	0 (0.0)	1 (10.0)	0 (0.0)	3 (30.0)	0 (0.0)	0 (0.0)
Tainan metropolitan area (N = 14)	0 (0.0)	0 (0.0)	0 (0.0)	2 (14.3)	0 (0.0)	0 (0.0)	0 (0.0)	2 (14.3)	0 (0.0)	0 (0.0)
Kaohsiung metropolitan area (N = 28)	2 (7.1)	1 (3.6)	3 (10.7)	2 (7.1)	4(14.3)	3 (10.7)	4 (14.3)	1 (3.6)	2(7.1)	1 (3.6)
Rural townships area (N = 167)	3 (1.7)	7 (4.0)	3 (1.7)	7 (4.0)	4 (2.3)	6 (3.5)	2 (1.2)	6 (3.5)	1 (0.6)	6 (3.5)
Aboriginal townships area (N = 55)	0 (0.0)	5 (9.1)	1 (1.8)	4 (7.3)	1 (1.8)	4 (7.3)	2 (3.6)	2 (3.6)	1 (1.8)	4 (7.3)
										
Total (N = 352)^c^	6 (1.7)	17 (4.8)	7 (2.0)	17 (4.8)	9 (2.6)	18 (5.1)	9 (2.6)	17 (4.8)	4 (1.1)	16 (4.5)

a. High-high association (hot spot), meanings utilization was higher than average

b. Low-low association (cold spot), meanings utilization was lower than average

c. Three areas did not presented any hot spot or cold spot including Taichung-Changhwa metropolitan, Chungli-Taoyuan metropolitan, and Chiayi secondary metropolitan areas.

d. Group A, agents acting on the rennin-angiotensin system; Group B, beta blocking agents; Group C, calcium channel blockers; Group D, diuretics; Group O, antihypertensives

By drug class, both Group C and Group D had the highest amount of diverse clusters across the country, followed by Group B, Group A, and Group O. Hot spot areas concentrated mostly in the Kaohsiung and rural areas among all drug classes (Table 2). In contrast, cold spot areas showed up in rural and aboriginal townships areas, accounting for a particularly high portion of group A, followed by group B, group C, group O, and group D.

## Discussion

Our study showed a variability of cardiovascular prescription patterns with regard to geographic differences in Taiwan. The analysis of both HH and LL associations strongly indicated that there exists a discrepancy in drug use between the north and the south of Taiwan, and the southern areas exhibited spatial clustering of unusual utilization in cardiovascular drugs. There are two possible explanations for this finding. First, the disease prevalence may be different between the north and south in Taiwan as a result of urbanization levels. The prevalence of cardiovascular disease, such as hypertension and coronary heart disease, varies between the urban and rural areas. Some countries reported the prevalence was higher for people living in the urban than rural areas [31,32], while others, including Taiwan, presented evidence to the contrary [33,34]. Second, there were urban-rural differences in the appropriateness of disease treatment and the inclination of patients to receive medical care. Previous studies have documented regional variation in quality of care in cardiovascular disease [22,35]. Patients hospitalized for acute myocardial infarction (AMI) in rural hospitals were less likely to receive recommended intervention than in urban hospitals. In addition, inappropriate or lower utilization of health care could be associated with lower socioeconomic status of patients in rural areas [36,37].

Utilization of cardiovascular drugs appeared to be associated with different classes of pharmacological drugs among geographic regions. We found that underutilization of all classes of drugs was more likely to be observed than overutilization, and the former was scattered mainly in the southern, rural, and aboriginal townships. (Figure 2) It seemed to suggest a wide variation in prescribing behavior. Drug Group A, Group B, and Group C were generally the most widely used drugs for the treatment of cardiovascular diseases [38]; however, there were large variations across townships with regard to the use of drugs. A study conducted in France reported the socio-economic status at the area level influenced the utilization of specialty care [39]. Differences between general practitioners and other specialties have been described in relation to the use of cardiovascular medicines [40-42]. For example, compared with cardiologists, family practitioners and general internists probably underutilize ACE inhibitors due to less knowledge about heart failure and poor adherence to guidelines [42]. Among seventeen townships where ACE inhibitors were underutilized, we found that a clinic for primary care is the major medical resource in these areas [43]. In addition, there are three aboriginal townships have the High-High hotspot. They tend to over-utilize Group B, C, D and O but not ACEI inhibitors. This might be attributable to the fact that the aboriginal residents only have access to general practitioners and medical treatment by specialists is lacking. Accordingly, the spatial patterns of drug use observed in this study suggested that there was a wide variation in physicians' prescribing behavior across Taiwan, to the extent that medical resources were not consistently utilized among 352 townships.

Utilization of cardiovascular drugs also associated with the disease patterns appeared to be different between townships. Some studies showed that rural residents are at higher risk for the burden of major diseases such as cardiovascular disorder and the severity of their complications [33,44]. In contrast, other studies showed that higher prevalence rates of cardiovascular risk factors were found in urban than rural areas due to diet and stressful life styles [31,45]. Drug choice should be based on clinical features of patients. For instance, rennin-angiotensin system agents have greatly improved clinical outcomes in patients with renal complications, [46] or diuretics are first-line agents for elderly hypertensive patients without other complications [47].

Spatial analytical methods recognize that spatial concordance between two proximal entities is closely connected by intrinsic causal relationship or hidden confounders. Although the geographical variations in drug use were widely documented, the concept of spatial dependence has received little attention in pharmacoepidemiology. It was our intention in this study to establish that "spatial perspectives in pharmacoepidemiology" would be an effective method in detecting the variation of drug utilization. That is, unusual drug prescriptions are not randomly located across locations but tend to be concentrated within certain areas.

LISAs are simply local derivations or disaggregation of global measures of spatial autocorrelation, and allow one to detail the information of local variations in spatial autocorrelation (e.g., hot spots and cold spots) for further study consideration[48]. This method is useful to indicate spatial correlation of similar values at location *i *and its neighborhood. This means that location *i *and its neighborhood can both have values above the average value (hot spots), or both can have values below the average (cold spots). Such local variations in spatial autocorrelation are statistically significant. Departure from the classical assumption of statistical independence, the spatial clusters represent that data are not randomly distributed over the global region and can occur because there is a contagious factor to the underlying process that is commonly shared by the clustered region. Lack of the factor that triggers spatial dependence does not give rise to spatial clustering.

The primary strength of this study was that we incorporated GIS and tools for spatial analysis to explore the role of geography in drug utilization. In so doing, we were able to divert our examination from the traditional framework, which only considers individual decision-making, into the spatial realm. Epidemiologic investigations are commonly involved with data from contiguous or proximal geographic areas, where neighboring locations may share similar prescribing patterns due to, for example, inhabitants' lifestyle behaviors or environmental exposures. Although the trend in utilization of cardiovascular drugs in Taiwan was investigated, little was known regarding its geographical pattern across townships. The spatial analysis presented here may add essential information to pharmacoepidemiological studies and policy-making. On the one hand, spatial analysis allows us to examine spatial processes underlying the prescribing behavior of physicians and provides the first inquiry into whether spatial components should be accounted for in modeling to avoid erroneous inference [49]. On the other hand, given the limited resources, the government can focus their policy efforts to target areas to improve drug utilization. For the reasons described above, our approach should be applicable worldwide. Developing countries, for which medical resources are increasingly disproportionately distributed, may see more distinct prescribing patterns than those for Taiwan or other developed countries.

Although spatial analysis with Moran's *I *is useful for this study, it does have limitations of which all should be aware. First, Moran's *I *(and other global indicators) is not powerful enough to differentiate well between a random pattern and a pattern without substantial spatial variations. Second, Moran's *I *is conditional, tied to how we define localities through a spatial weights matrix *w*_*ij*_. Different definitions of the matrix can lead to different values of spatial dependence. Finally, the measures of spatial dependence are subject to the modifiable areal unit problem. The problem arises due to the arbitrary choice and the modifiable nature of areal units in spatial analysis, in the sense that they can be aggregated to form units of different sizes of spatial arrangements. The degree of spatial association, as measured by Moran's *I*, changes with different levels of aggregation in areal units. While we are aware of the limitations of the spatial autocorrelation index, our results in this paper can be interpreted as a broad indication of the presence and magnitude of spatial dependence.

Other potential limitations of our study also should be noted. First, the utilization pattern of cardiovascular medications could be different as urbanization changes. Second, we only analyzed the classes of cardiovascular drugs based on the first three digits of ATC which have DDD assigned. Although we were able to depict the spatial patterns of drug use, whether the knowledge of new drugs was attributable to the geographic clustering requires a detailed classification of drug classes based on ATC. Third, we might underestimate the utilization of group A, B, D because complex medication (i.e., a compound made of two or more ingredients) without DDD assigned were not estimated in this study. The most complex medication are combinations with group D (i.e., group A plus group D or group B plus group D) to improve patient's compliance. Fourth, this study only analyzed one year of data, but the prescribing pattern could extend across years. Fifth, the cardiovascular medication pattern did not correspond to disease distribution. Most patients need two more classes of medication to control their disease, except simple essential hypertension[47]. Finally, the fact that we have observed significant spatial autocorrelation in our data does not determine whether these are true spatial effects or are spurious in the sense that they can be attributed entirely to patterns in other variables, such as income or individual characteristics (such as age, gender, etc). If we control for all of these other factors and the spatial variable remains significant, then we have evidence that the patterns are consistent with a neighborhood effect and are not solely attributable to socioeconomic characteristics of these areas. Exploring theses sources of spatial patterns and the mechanisms that may be driving them is an obvious extension of the analysis presented here, and more detailed studies are necessary to better understand the factors that influence prescribing patterns.

## Conclusions

The cardiovascular medication prescribing patterns were heterogeneous across Taiwan. In particular, a clear pattern of north-south disparity exists. The spatial variability of prescribing patterns documented in this study shows that priority-setting in research may heavily depend on the neighborhood association.

## List of abbreviations

AMI: Acute myocardial infarction; ATC: Anatomical Therapeutic Chemical; CVD: Cardiovascular Disease; GIS: Geographic Information Systems; LISA: Local Indicators of Spatial Association; NHIRD: National Health Insurance Research Database; WHO: World Health Organization

## Competing interests

The authors declare that they have no competing interests.

## Authors' contributions

CLC, YCC, TML, and YHKY designed the study. CLC and TML analyzed the data. CLC and YCC drafted the initial article. All authors were involved in critical evaluation and editing of the manuscript and read and approved the final version.

## Pre-publication history

The pre-publication history for this paper can be accessed here:

http://www.biomedcentral.com/1471-2458/11/380/prepub
